# Screening ovarian cancer by using risk factors: machine learning assists

**DOI:** 10.1186/s12938-024-01219-x

**Published:** 2024-02-12

**Authors:** Raoof Nopour

**Affiliations:** Department of Health Information Management, Student Research Committee, School of Health Management and Information Sciences Branch, Iran University of Medical Sciences, Tehran, Iran

**Keywords:** Machine learning, Public health challenge, Predictive efficiency, Ovarian cancer, Preventive strategy

## Abstract

**Background and aim:**

Ovarian cancer (OC) is a prevalent and aggressive malignancy that poses a significant public health challenge. The lack of preventive strategies for OC increases morbidity, mortality, and other negative consequences. Screening OC through risk prediction could be leveraged as a powerful strategy for preventive purposes that have not received much attention. So, this study aimed to leverage machine learning approaches as predictive assistance solutions to screen high-risk groups of OC and achieve practical preventive purposes.

**Materials and methods:**

As this study is data-driven and retrospective in nature, we leveraged 1516 suspicious OC women data from one concentrated database belonging to six clinical settings in Sari City from 2015 to 2019. Six machine learning (ML) algorithms, including XG-Boost, Random Forest (RF), J-48, support vector machine (SVM), K-nearest neighbor (KNN), and artificial neural network (ANN) were leveraged to construct prediction models for OC. To choose the best model for predicting OC, we compared various prediction models built using the area under the receiver characteristic operator curve (AU-ROC).

**Results:**

Current experimental results revealed that the XG-Boost with AU-ROC = 0.93 (0.95 CI = [0.91–0.95]) was recognized as the best-performing model for predicting OC.

**Conclusions:**

ML approaches possess significant predictive efficiency and interoperability to achieve powerful preventive strategies leveraging OC screening high-risk groups.

## Introduction

Ovarian cancer (OC) is ranked seventh and eighth with regard to tumor malignancy prevalence and death among women globally [1]. They rank third in mortality after uterine and cervical as gynecological cancers [2]. This cancer usually emerges from ovarian epithelial cells in the ovary. It is frequently diagnosed at advanced stages due to poor prognosis and a lack of more appropriate screening test solutions [3, 4]. The mysterious progression and the high prevalence of OC among women have imposed a public health challenge [5]. OC caused 240,000 new cases worldwide and accounts for the second incidence of cancer following breast in women [6, 7]. The OC sickens 22,000 new cases and causes 14,000 mortalities in the United States annually [8]. The risk of OC would be raised by increasing age, family history, changing genes, or family history of the syndrome among women; in contrast, some determinants such as contraceptive pills consumption, oophorectomy, and increasing parity have the preservative role in OC development [9, 10]. Despite the high prevalence of OC worldwide, in some developed countries, the incidence of the disease has diminished to some extent due to the mentioned supportive factors and suitable preventive and early detection strategies in recent decades [11, 12]. However, variation associated with OC risk exists worldwide; the Asian, Central and Eastern European, and Central and South American countries account for high-risk regions in terms of OC incidence [13]. It is estimated that OC incidence and death rates will increase worldwide by 2035, requiring better judgment by health policymakers, especially for women older than 65 and those living in regions lacking preventive or therapy services [14]. In Iran, the OC has the eighth prevalence rank among neoplasms, with a 61% five-year survival rate. Iran had 1966 and 1269 new cases and a mortality rate of OC among women in 2020 [15]. Despite the increasing trend of OC among women due to the decreasing birth rate and increasing elderly population, it has not been suggested as an effective solution for screening this disease [16, 17]. OC would be detected at advanced stages due to the asymptomatic nature of this disease at earlier stages, and even differential diagnosis to other maladies at later stages, leading to poor prognosis [18].

Although some aggressive methods exist for screening high-risk OC women, such as removing small sections of the uterus, we require a more effective preventive strategy due to the high false positive results rate associated with existing screening methods [19]. Machine learning (ML) is a subfield of artificial intelligence (AI) that leverages past data to build knowledge structures and learn from data to predict future events based on these structures achieved by past data [20]. Leveraging ML has significantly promoted the therapy, medication, diagnosis, prediction, and screening of medical conditions such as cancer [21, 22]. Past research has shown that ML-based approaches can provide practical cancer screening through high-performing risk prediction [23, 24].

Some recently invented ML algorithms indicated significant predictive capability concerning various biomedical topics. For example, iMethyl-STTNC is recognized as an effective technique in the detection of methyladenosine sites in RNA [25]. iACP-GAEnsC' model as an evolutionary genetic algorithm-based ensemble approach gained efficient predictive capability in anticancer peptides classification [26]. DP-binder plays a crucial role in different biological processes, including rejoining, replicating, and repairing DNA [27]. iHBP-DeepPSSM is considered an accurate and reliable technique for the identification of hormone-binding proteins [28]. Other ML approaches, including "iAtbP-Hyb-EnC" and the cACP-DeepGram model, are leveraged in cancer therapy and suggested as a fruitful ensemble technique in academic study and drug discovery [29, 30].

One branch of ML is deep learning (DL), which uses particular artificial neural network configurations to efficiently learn from more sophisticated data such as images, sounds, signals, etc. [31]. Despite this approach, the ML has the potential to perform best in structured databases that possess low and medium volume [32, 33]. Based on investigating past works on leveraging ML and DL in the risk prediction of OC, no study was conducted on this topic. Studies are conducted on screening the OC in the early stages of this disease or predicting OC using malignant and benign cases [34, 35]. Therefore, this study aims to introduce a screening solution based on risk factors and an ML approach to stratify high-risk and low-risk people as a preventive strategy. To this aim, we first gathered the data on this topic and prepared it for mining purposes. In the preparation process of data, we use three strategies: eliminating the data redundancy, embedding the missing values, and selecting the best factors concerning prediction purposes. Then, we leverage ML algorithms based on the enhanced data and stratified factors to build the prediction model on this topic. Based on the various feature importance techniques, we assess all factors influencing the OC prediction in an explainable way. Previous studies leveraged this process to build the prediction model for various biomedical purposes. Afrash et al. used Minimum Redundancy Maximum Relevance (mRMR) feature selection with the ensemble and non-ensemble ML algorithms to diagnose COVID-19 based on clinical data [36]. Shanbehzadeh et al. leveraged ML algorithms and preprocessing steps for breast cancer as a single-centered study approach [37]. They concluded that using the ML techniques plays a significant role in prediction strategy. Nopour et al. developed a prediction model for the mortality of COVID-19 patients based on statistical and computational ML techniques and phi-coefficient as a feature selection process [38]. Nopour et al. assessed various configurations of ANNs to design an intelligent tool for breast cancer prognosis. This study used the Chi-square as a feature selection technique in one single-centered study [39].

## Results

### Preprocessing database

**Fig. 1:**
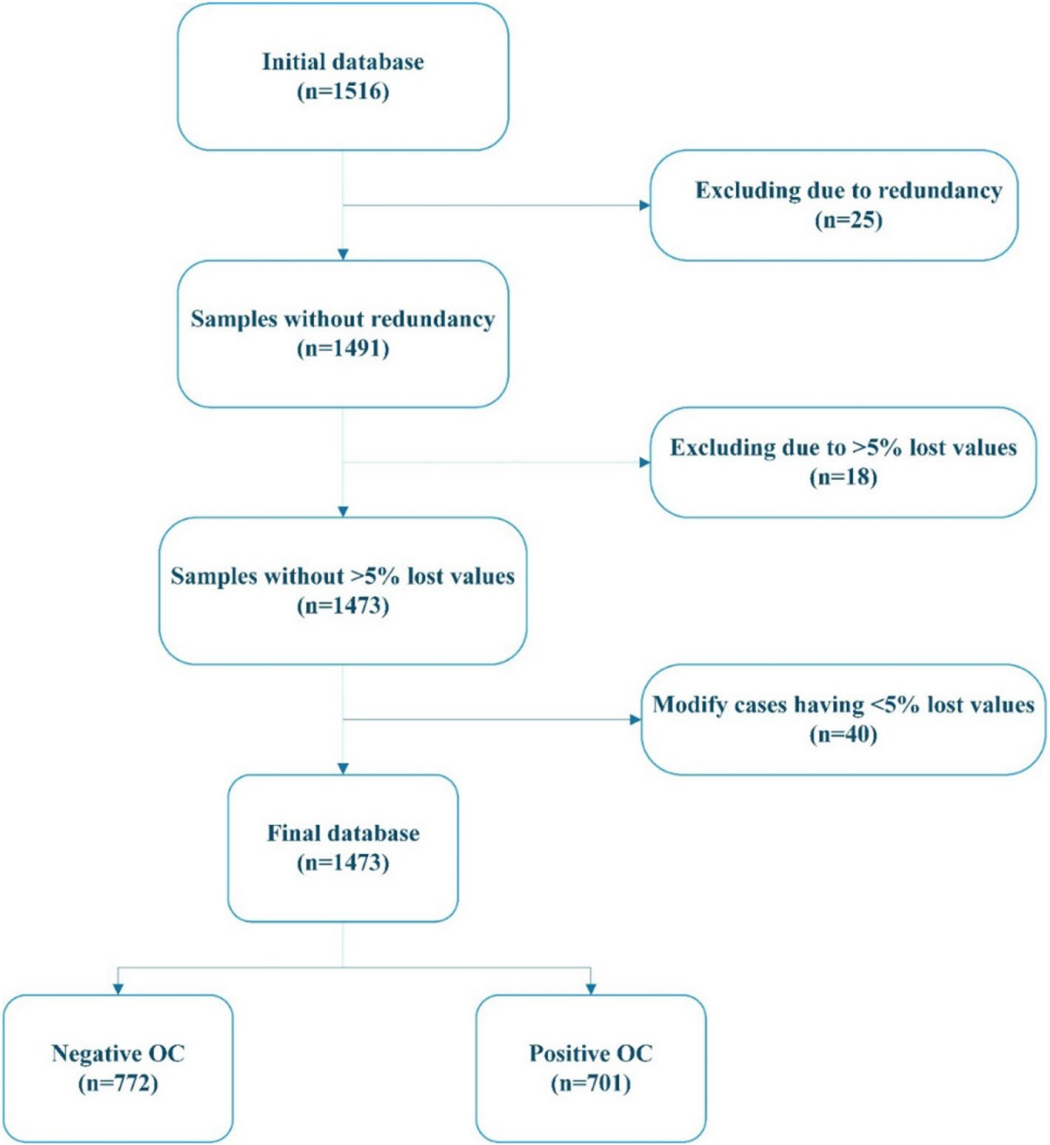
The preprocessing steps of the samples in the dataset

After investigating the database, some redundant cases were identified; this sameness originated from different identification numbers (IDs) for the same person when integrating databases due to a lack of interoperability between these centers. Thereby, 25 duplicated records, including seven and 18 cases associated with positive and negative cases, respectively, were excluded from the study. Reviewing the database concerning lost values, we discovered that 18 cases, including five and 13 cases belonging to positive and negative, possess more than 5% missing values. So, we removed them from the study. Also, the values of 40 records with less than 5% missing data were imputed using the KNN algorithm. This way, the replacement methods using predictive algorithms have less bias than other methods, such as using values having the highest frequency, etc.; therefore, model effectiveness concerning generalizability will be maintained to a large extent. Finally, 1473, including 701 and 772 cases belonging to positive and negative cases, remained in the current study, as Fig. 1 shows.

The characteristics of the samples among positive and negative OC groups are presented in Table 1.

**Table 1:** OC suspicious characteristics of samples

Feature	Value	Total frequency (*n* = 1473)	Positive	Negative
			**(*n* = 701)**	**(*n* = 772)**
Age	< 40 40–50 50–60 > 60	205 424 617 227	85 175 320 121	120 249 297 106
BMI	< 18.5 18.5–25 25–30 > 30	57 311 475 630	25 107 198 371	32 204 277 259
Blood group	A B AB O	598 257 298 320	339 102 123 137	259 155 175 183
Race	Persian Others	1150 323	519 182	631 141
Menopausal age	< 45 45–50 50–55 > 55	52 208 655 558	23 71 349 258	29 137 306 300
Postmenopausal hormone therapy	Yes No	845 628	459 242	386 386
Endometriosis	Yes No	743 730	472 229	271 501
History of nonpregnancy	Yes No	805 668	459 249	346 419
Family history of cancer such as ovary, breast, or colorectal	Yes No	859 614	466 235	393 379
Family cancer syndrome	Yes No	375 1098	212 489	163 609
Fertility treatment use	Yes No	569 904	273 428	296 476
Breast cancer	Yes No	456 1017	277 424	179 593
Smoking	Yes No	345 1128	211 490	134 638
History of pregnancy and breastfeeding before age 26	Yes No	779 694	318 383	461 311
History of the PCOS	Yes No	597 876	302 399	295 477
History of chest X-ray	Yes No	658 815	389 312	269 503
Alcohol consumption	Yes No	52 1421	25 676	27 745
Particular food consumption, such as fried foods, whole milk, and trans fats	Yes No	852 621	596 105	256 516
History of exposure to mutagenic or chemical substances	Yes No	398 1075	202 499	196 576
High red meat consumption	Yes No	412 1061	184 517	228 544
History of hysterectomy	Yes No	358 1115	274 427	84 688
Oral contraceptive pill use	Yes No	474 999	167 534	307 465
Aspirin use	Yes No	872 601	287 414	585 187
High consumption of coffee	Yes No	257 1216	122 579	135 637
Vegetable consumption	Low Medium High	504 530 439	235 269 197	269 261 242
Fruit consumption	Low Medium High	426 518 529	243 249 209	183 269 320

### Feature selection

The results of determining the correlation of predictors associated with OC using MLR are shown in Table 2.

**Table 2:** Analysis of OC predictors using MLR

Feature	*β*	OR	95% CI of OR	*P*_-value_
Age	0.521	1.275	[1.215–1.472]	**0.01**
BMI	0.334	1.179	[1.079–1.343]	**0.01**
Blood group	0.188	1.121	[1.053–1.278]	**0.03**
Race	0.052	0.927	[0.892–1.148]	0.1
Menopausal age	0.294	1.03	[1.012–1.196]	**0.04**
Postmenopausal hormone therapy	0.255	1.24	[1.191–1.334]	**0.01**
Endometriosis	0.451	1.645	[1.572–1.837]	** < 0.001**
History of nonpregnancy	0.674	1.994	[1.727–2.446]	** < 0.001**
Family history of cancer such as ovary, breast, or colorectal	0.319	1.274	[1.256–1.349]	**0.01**
Family cancer syndrome	0.118	1.032	[1.011–1.056]	**0.045**
Fertility treatment use	0.072	0.958	[0.873–1.156]	0.07
Breast cancer	0.174	1.056	[1.023–1.103]	**0.04**
Smoking	0.293	1.155	[1.093–1.257]	**0.03**
History of pregnancy and breastfeeding before age 26	0.252	1.089	[1.036–1.157]	**0.04**
History of PCOS	0.378	1.526	[1.455–1.724]	**0.01**
History of chest X-ray	0.412	1.256	[1.181-0.1.324]	**0.02**
Alcohol consumption	0.163	1.163	[0.776–1.554]	0.165
Particular food consumption, such as fried foods, whole milk, and trans fats	0.434	2.016	[1.774–2.347]	** < 0.001**
History of exposure to mutagenic or chemical substances	0.062	0.974	[0.665–1.257]	0.12
High red meat consumption	0.126	1.072	[0.824–1.123]	0.08
History of hysterectomy	0.538	1.986	[1.795–2.623]	** < 0.001**
Oral contraceptive pill use	− 0.473	0.512	[0.345–0.679]	** < 0.001**
Aspirin use	− 0.225	0.498	[0.452–0.667]	**0.01**
High consumption of coffee	0.16	0.773	[0.572–1.231]	0.13
Vegetable consumption	0.075	0.892	[0.652–1.453]	0.185
Fruit consumption	0.09	0.805	[0.452–1.375]	0.123

*β*: correlation, OR: odd ratio, CI: confidence interval

As shown in Table 2, based on the MLR, the factors including age (*β* = 0.521, OR = 1.275, 95% CI  [1.215–1.472]), BMI (*β* = 0.334, OR = 1.179, 95% CI  [1.079–1.343]), blood group (*β* = 0.188, OR = 1.121, 95% CI  [1.053–1.278]), menopausal age (*β* = 0.294, OR = 1.03, 95% CI  [1.012–1.196]), postmenopausal hormone therapy (*β* = 0.255, OR = 1.24, 95% CI  [1.191–1.334]), endometriosis (*β* = 0.451, OR = 1.645, 95% CI  [1.572–1.837]), family history of cancer such as ovary, breast, or colorectal (*β* = 0.319, OR = 1.274, 95% CI  [1.256–1.349]), family cancer syndrome (*β* = 0.118, OR = 1.032, 95% CI  [1.011–1.056]), breast cancer (*β* = 0.174, OR = 1.056, 95% CI  [1.023–1.103]), smoking (*β* = 0.293, OR = 1.155, 95% CI  [1.093–1.257]), history of pregnancy and breastfeeding before age 26 (*β* = 0.252, OR = 1.089, 95% CI  [1.036–1.157]), history of PCOS (*β* = 0.378, OR = 1.526, 95% CI  [1.455–1.724]), history of chest X-ray (*β* = 0.412, OR = 1.256, 95% CI  [1.181–1.324]), particular food consumption, such as fried foods, whole milk, and trans fats (*β* = 0.434, OR = 2.016, 95% CI  [1.774–2.347]), history of hysterectomy (*β* = 0.538, OR = 1.986, 95% CI  [1.795–2.623]), oral contraceptive pill use (*β* =  particular food consumption 0.473, OR = 0.512, 95% CI  [0.345-0.679]), and aspirin use (*β* = − 0.225, OR = 0.498, 95% CI  [0.452–0.667]) were considered as the essential factor associated with OC prediction at *P* < 0.05. In contrast, other predictors including race, fertility treatment use, alcohol consumption, history of exposure to mutagenic or chemical substances, high red meat consumption, high consumption of coffee, vegetable consumption, and fruit consumption did not gain significance over 95% confidence, thereby excluded from the study (P > 0.05).

### Model development and assessment

The results of measuring the ML-trained algorithms' performance, along with best-adjusted hyperparameters for development by grid search, are presented in Tables 3 and 4. The ranges of hyperparameters used for training the ML algorithms are presented in Table 5.

**Table 3:** The results of ML-trained performance

Algorithm	PPV	NPV	Sensitivity	Specificity	Accuracy	F-score
ANN	0.75 ± 0.035	0.82 ± 0.027	0.81 ± 0.038	0.75 ± 0.021	0.78 ± 0.029	0.78 ± 0.03
KNN	0.70 ± 0.029	0.74 ± 0.026	0.72 ± 0.032	0.72 ± 0.022	0.72 ± 0.027	0.71 ± 0.029
J-48	0.71 ± 0.043	0.75 ± 0.037	0.73 ± 0.046	0.73 ± 0.035	0.73 ± 0.038	0.72 ± 0.039
RF	0.89 ± 0.021	0.89 ± 0.016	0.88 ± 0.028	0.90 ± 0.012	0.89 ± 0.021	0.88 ± 0.023
SVM	0.72 ± 0.033	0.77 ± 0.025	0.76 ± 0.036	0.73 ± 0.02	0.74 ± 0.028	0.74 ± 0.03
XG-Boost	0.94 ± 0.015	0.93 ± 0.005	0.93 ± 0.019	0.95 ± 0.002	0.94 ± 0.008	0.94 ± 0.01

**Table 4:** Best hyperparameters tuned

Algorithm	Hyperparameter
ANN	Number of hidden layers: 8; learning rate: 0.8; training epoch: 100; validation threshold: 50; nominal to binary filter: true
KNN	3 ≤ K ≤ 7; Nearest neighbor search algorithm: Euclidean; Distance weighting: 1/distance
J-48	Binary split: false; number of objects: 2; confidence factor: 0.3; reduced pruning: true; number of folds: 3; Use Laplace: true
RF	Max_Depth: 8; number of iterations: 100; calculate out of bag: true; number of randomly chosen features: 6; classifiers: decision stump
SVM	Kernel type: RBF; calibrator: logistic; Epsilon: 1.0E−12; c:10; tolerance parameter: Num folds: − 1; RBF-gamma: 0.1
XG-Boost	Booster: gb-tree; nthread: MAX; eta: 0.5; Gamma: 1; max_depth: 8; mi_child_weight: 1; max delta step: 0; sub_sample:1; Lambda:1; alpha: 0; scale_pos_weight: 1; objective: binary:logistic

RBF: radial basis function

**Table 5:** The ranges of hyperparameters used for training ML algorithms

Algorithm	Ranges of parameters used as grid-search technique
ANN	Number of hidden layers [5,20]; learning rate [0.3,1]; validation threshold [20,100]
KNN	K [3,7]
J-48	Number of objects [1,5]; confidence factor [0.15,0.45]; number of fold [2,6]
RF	MAX_Depth [6,20]; number of randomly chosen features [5,20]
SVM	c [1,100]; RBF-gamma [0.1,1]
XG-Boost	Eta [0.3,1]; Gamma [0,2]; max_depth [5,20]; min_child_weight [0,5]

**Fig. 2:**
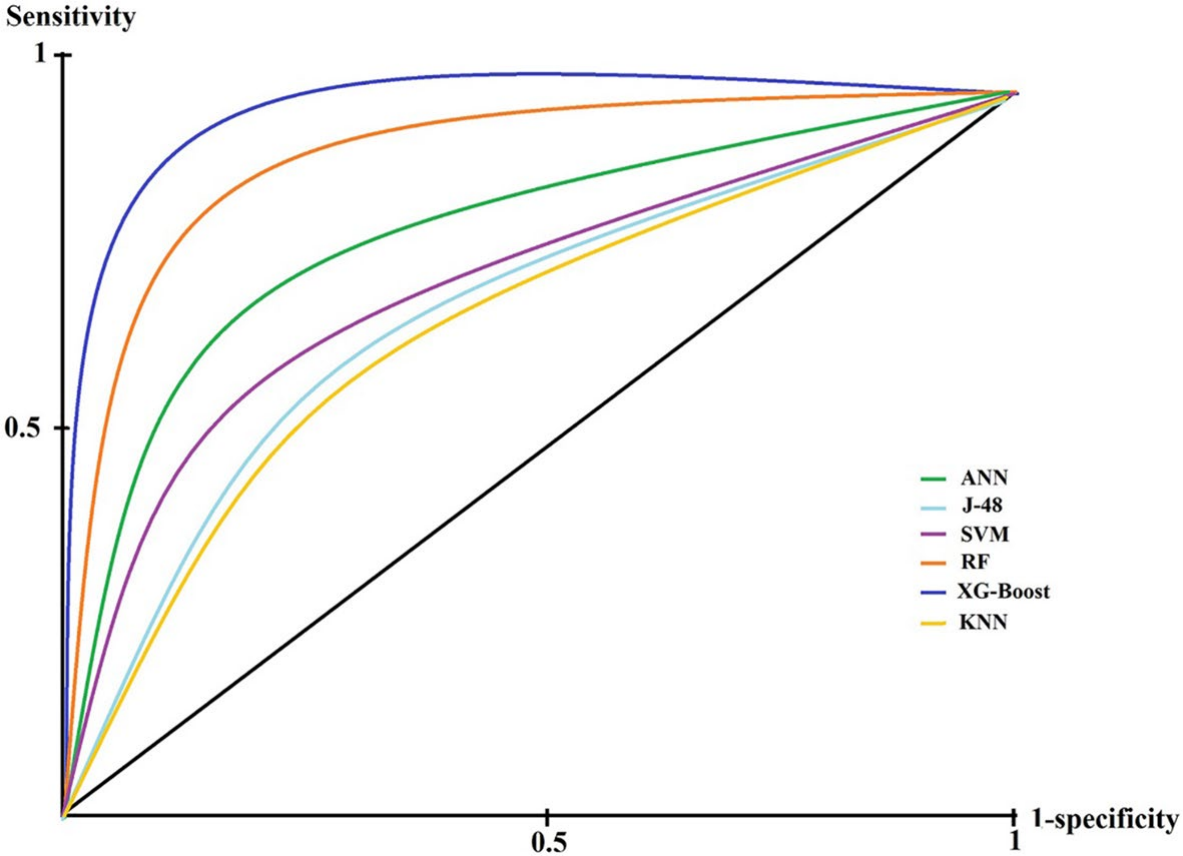
The ROC of ML-trained algorithms

As presented in Tables 3 and 4, the ANN-trained algorithm with 15 hidden layers and a 0.8 learning rate obtained with the maximum epoch of 100 when training obtained PPV = 0.75 ± 0.035, NPV = 0.82 ± 0.027, sensitivity = 0.81 ± 0.038, specificity = 0.75 ± 0.021, accuracy = 0.78 ± 0.029, and F-Score = 0.78 ± 0.03. KNN gained PPV = 0.70 ± 0.029, NPV = 0.74 ± 0.026, sensitivity = 0.72 ± 0.032, specificity = 0.72 ± 0.022, accuracy = 0.72 ± 0.027, and F-Score = 0.71 ± 0.029 with K between 3 to 7 and Euclidean as the distance scale. J-48 with 0.3 confidence factor, had PPV = 0.71 ± 0.043, NPV = 0.75 ± 0.037, sensitivity = 0.73 ± 0.046, specificity = 0.73 ± 0.035, accuracy = 0.73 ± 0.038, and F-Score = 0.72 ± 0.039. RF achieved a performance of PPV = 0.89 ± 0.021, NPV = 0.89 ± 0.016, sensitivity = 0.88 ± 0.028, specificity = 0.90 ± 0.012, accuracy = 0.89 ± 0.021, and F-Score = 0.88 ± 0.023 through max_depth tree of 8, decision stump as the classifier, and number of the randomly chosen tree of 6. SVM obtained PPV = 0.72 ± 0.033, NPV = 0.77 ± 0.025, sensitivity = 0.76 ± 0.036, specificity = 0.73 ± 0.02, accuracy = 0.74 ± 0.028, and F-Score = 0.74 ± 0.03 as using RBF kernel type, regularizer = 10, and RBF-gamma = 0.1. Finally, XG-Boost used eta = 0.05, gamma = 1, and a maximum tree depth of 8 as best-adjusted hyperparameters obtained PPV = 0.94 ± 0.015, NPV = 0.93 ± 0.005, sensitivity = 0.93 ± 0.019, specificity = 0.95 ± 0.002, accuracy = 0.94 ± 0.008, and F-Score = 0.94 ± 0.01. By looking at performance indicators results associated with chosen algorithms-trained, we concluded that the XG-Boost has higher sensitivity, specificity, accuracy, etc., and gained better prediction capability than other ML-trained algorithms for OC. In contrast, the KNN obtained less performance efficiency than others. Regardless, we do not satisfy these criteria to compare prediction capability. The area under the receiver characteristic operator curve (AU-ROC) will grant better insight into prediction capability contrasting aims. The ROC curve of all ML-trained algorithms is depicted in Fig. 2.

As it is noticeable from Fig. 2, the ROC belonging to the XG-boost algorithm is closer to sensitivity vertices than others. On the contrary, the KNN gained more distance from it. Based on Fig. 2, the XG-Boost model with AU-ROC = 0.93 (0.95 CI [0.91–0.95]) gained more capability than other ML-trained algorithms concerning OC prediction. RF-trained algorithm with AU-ROC = 0.87 (0.95 CI [0.84–0.89]) gained the second rank in this regard. The ANN (AU-ROC = 0.75 (0.95 CI [0.72–0.79])), SVM (AU-ROC = 0.68 (0.95 CI  [0.65–0.70])), and J-48 models (AU-ROC = 0.65 (0.95 CI = [0.62–0.69])) obtained the third, fourth and fifth places to predict OC, respectively. Finally, the KNN-trained algorithm with (AU-ROC = 0.62 (0.95 CI  [0.60–0.65])) was considered as the weakest ML-trained algorithm regarding OC prediction. Generally, based on the performance results obtained, we concluded that the XG-Boost-trained algorithm is the most efficient model for OC prediction. Another insight gained from comparing purposes was that the XG-Boost and RF models achieved the best performance capability concerning OC prediction; hence, the ensemble algorithms have more performance efficiency in predicting OC than other ML algorithms.

**Fig. 3:**
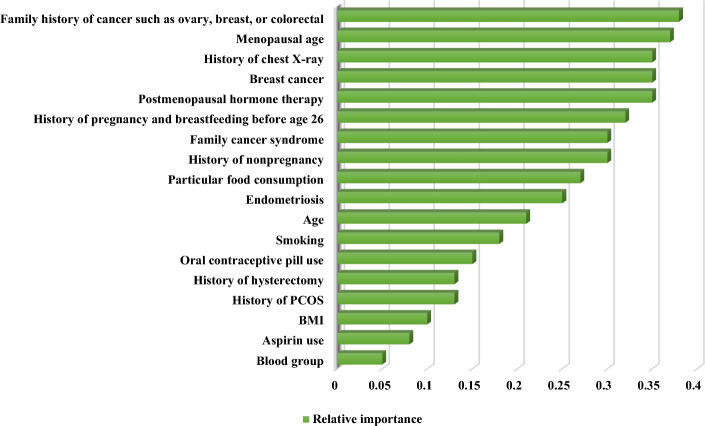
The RI of factors associated with OC prediction

We measured the predictors’ relative importance (RI) based on the XG-Boost as the best-performing algorithm. The results of the predictors’ RI are illustrated in Fig. 3.

**Fig. 4:**
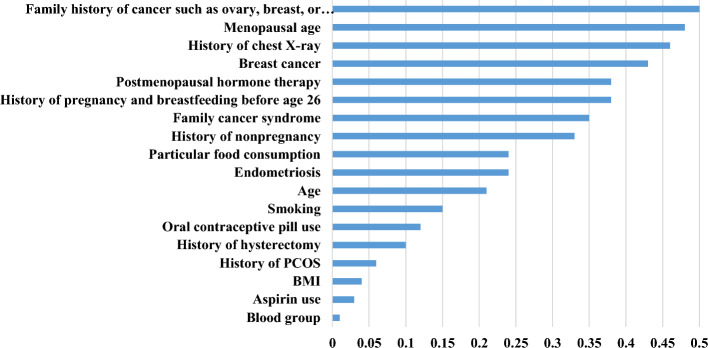
The importance of factors based on permutation feature score

**Fig. 5:**
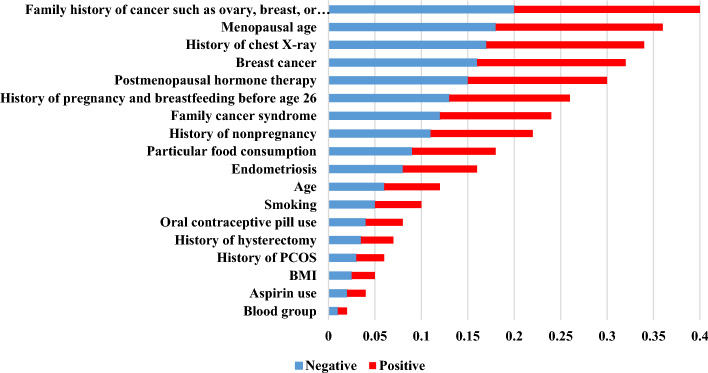
The mean SHAP values associated predictors of OC

**Fig. 6:**
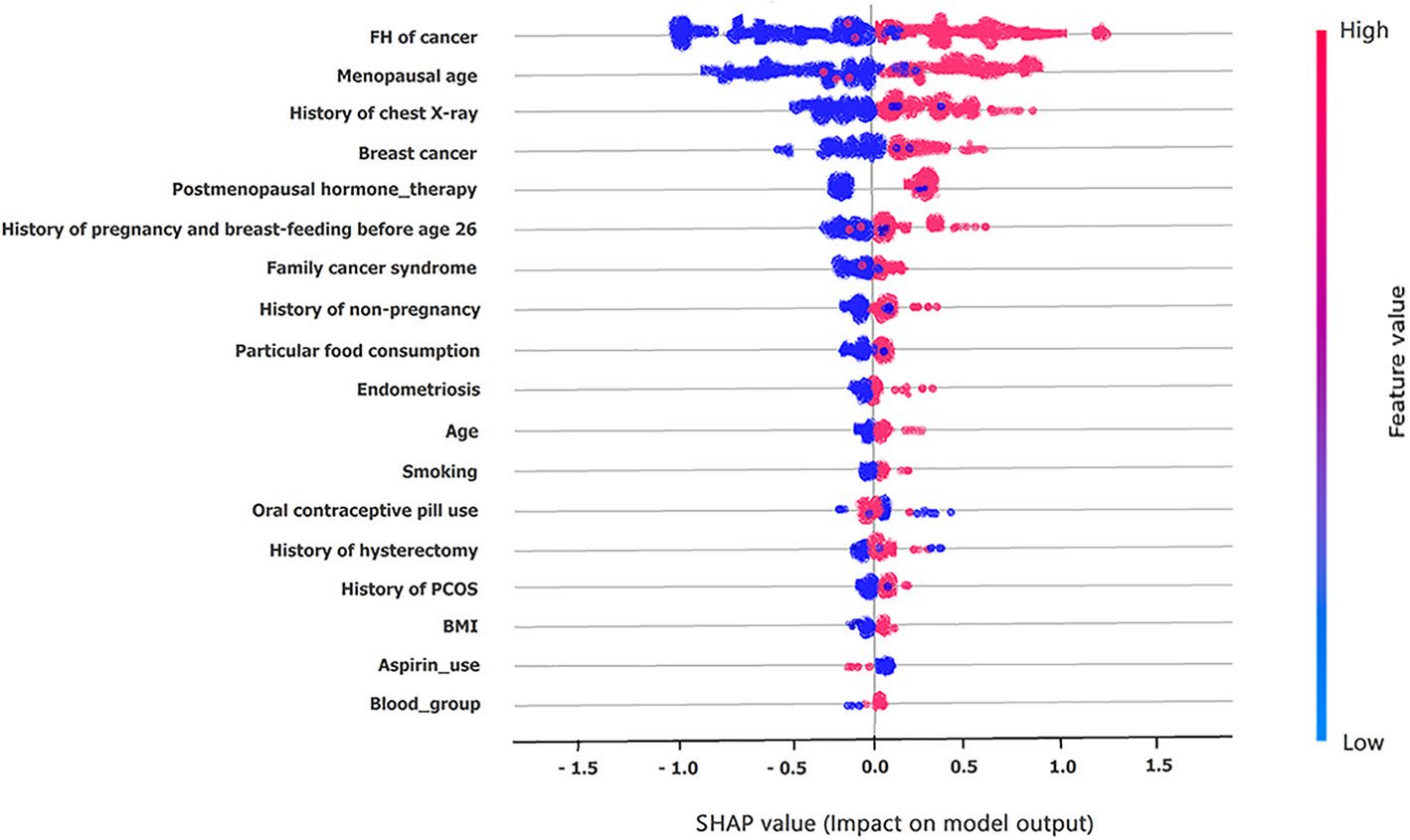
SHAP values associated with OC prediction pertaining to all cases

Based on Fig. 3, the predictors, including the family history of cancer such as ovary, breast, or colorectal (RI = 0.38), menopausal age (RI = 0.37), history of chest X-ray (RI = 0.35), personal history of breast cancer (RI = 0.35), and postmenopausal hormone therapy (RI = 0.35) gained more importance than others. They were considered the best predictors influencing OC prediction based on the XG-Boost model. On the contrary, factors such as blood group (RI = 0.1), BMI (RI = 0.08), and aspirin use (RI = 0.05) gave us less predictive insight concerning OC risk prediction based on XG-Boost. We also depicted the importance of the current predictors concerning OC based on the permutation feature score, mean SHapley Additive exPlanations (SHAP), and the SHAP values in Figs. 4, 5 and 6.

Based on the permutation feature score, the family history of cancer, such as ovary, breast, or colorectal, menopausal age, history of chest X-ray, personal history of breast cancer, and postmenopausal hormone therapy were considered as the best factors to predict OC. Also, based on the mean SHAP values and SHAP values pertaining to all OC cases, these factors were considered the most significant predictors concerning OC risk.

### External performance assessment

**Fig. 7:**
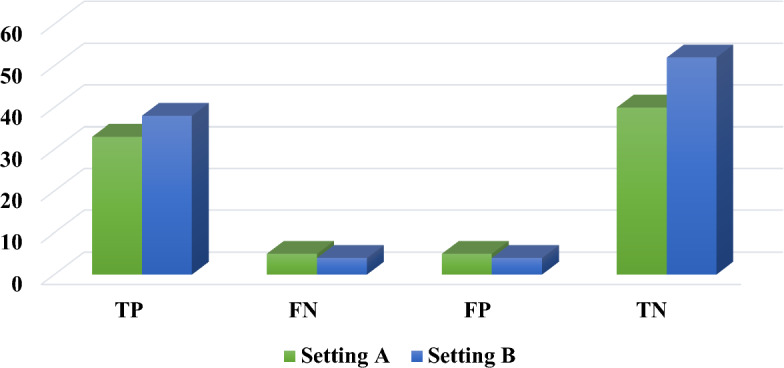
The external capability classification

As mentioned in the method section, we used the data from two external clinical settings to assess the generalizability capability of our best-performing model for predicting OC. Hence, we used 83 (38 and 45 positive and negative cases, respectively) and 98 (42 and 56 positive and negative cases, respectively) OC cases from these two clinical centers. The results of classifying the external data records by TN, FP, FN, and TN leveraging the XG-Boost model are shown in Fig. 7.

**Fig. 8:**
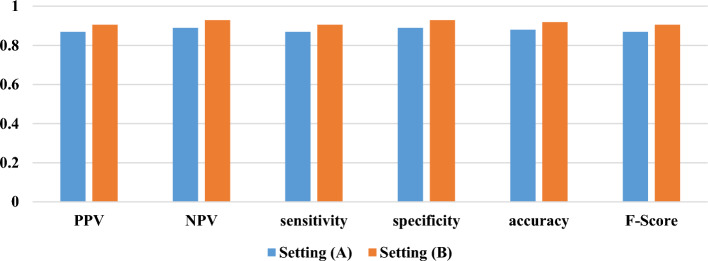
The performance indicators of XG-Boost in two external settings

As shown in Fig. 7, the XG-Boost model gained TP = 33, FN = 5, FP = 5, and TN = 40 and TP = 38, FN = 4, FP = 4, TN = 52 for settings A and B, respectively. The results of measuring the performance of the classified cases concerning two external clinical environments by XG-Boost are presented in Fig. 8.

As shown in Fig. 8, the XG-Boost gained PPV = 0.868, NPV = 0.888, sensitivity = 0.868, specificity = 0.888, accuracy = 0.879, and F-Score = 0.868 for the clinical external setting A, and also obtained PPV = 0.904, NPV = 0.928, sensitivity = 0.904, specificity = 0.928, accuracy = 0.918, and F-Score = 0.904 for setting B. By evaluating the prediction capability of the XG-Boost based on the external data cases, we observed that all the results of the performance indicators are in favorable state (> 0.8), indicating the pleasant generalizability of the current model built to predict OC risk among women.

**Fig. 9:**
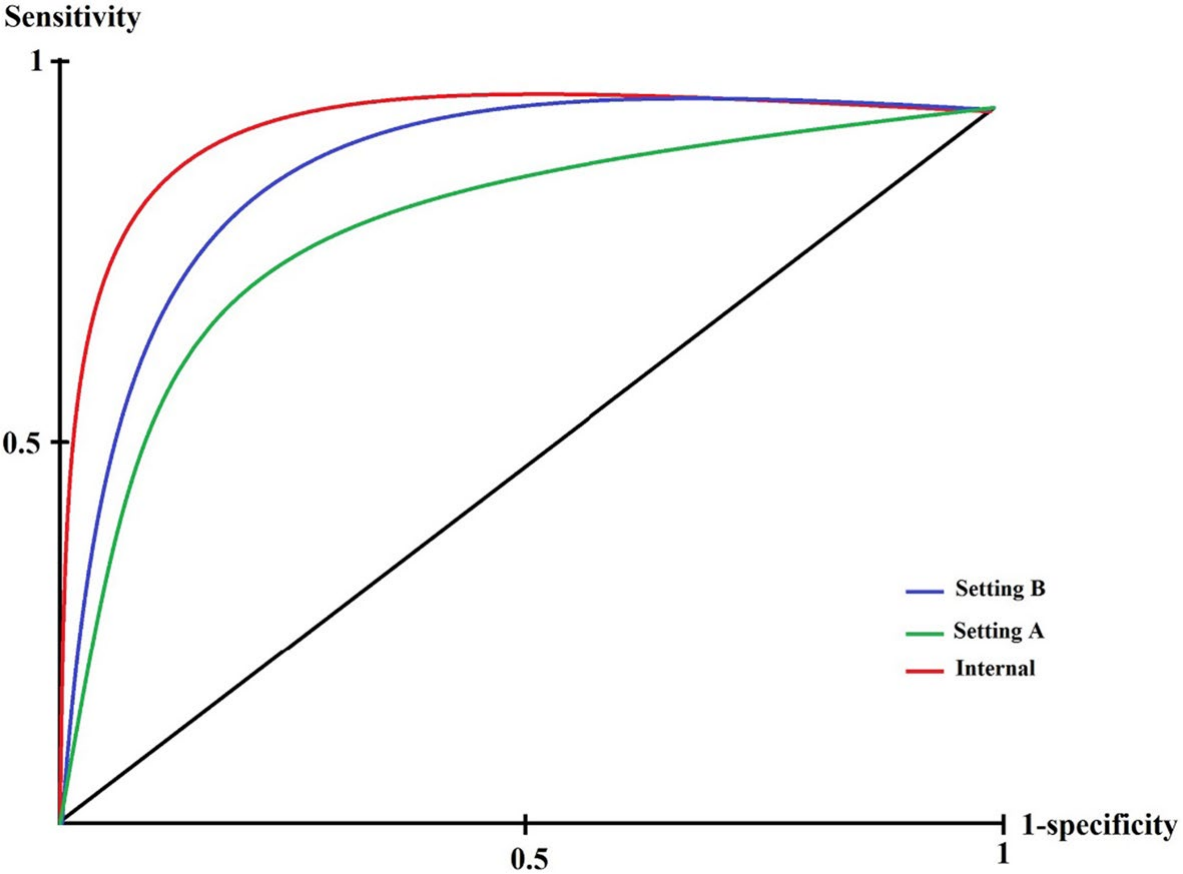
The internal and external ROC of the XG-Boost model

Also, based on the plotted ROC of the XG-Boost model when classifying external data cases to assess the OC prediction generalizability (Fig. 9), we obtained AU-ROC = 0.85 (0.95 CI [0.82–0.89]) and AU-ROC = 0.89 (0.95 CI [0.86–0.93]) for settings A and B, respectively, when classifying external data. By comparing the AUC-ROC of the XG-Boost model using internal data cases for the training model, AU-ROC = 0.93 (0.95 CI [0.91–0.95]) and AU-ROC of these two external settings, we noticed that the performance differences in the two states of internal and external modes were almost in a small amount (< 0.1 and < 0.05 AU-ROC for settings A and B, respectively) than the AU-ROC in internal state, indicating the pleasant comprehensiveness of the current ML model to predict OC risk.

## Discussion

Considering the increasing OC prevalence, especially in developing countries, and the mysterious nature of the OC progression, leveraging effective preventive strategies plays a significant role in decreasing the OC rate and their adverse outcomes and increasing the patient’s quality of life at the community level. So, this study aimed to get ML assistance as a potential predictive solution for screening OC based on risk factors. To this aim, we devised an ML data-driven approach; hence, we used a concentrated database belonging to six clinical centers associated with OC diagnosis. After preprocessing and preparing the database, we used chosen ML algorithms and fed them using OC positive and negative data to construct prediction models. Finally, the best ML-trained algorithm was chosen for prediction purposes with the highest performance in classifying the positive and negative OC cases. Also, the most influencing factors associated with OC prediction were extracted from the best-performing ML-trained algorithm. After gaining the best predicting model for OC, we tested its generalizability using data from two external clinical settings. The current study revealed that the XG-Boost model with PPV = 0.94 ± 0.015, NPV = 0.93 ± 0.005, sensitivity = 0.93 ± 0.019, specificity = 0.95 ± 0.002, accuracy = 0.94 ± 0.008, F-Score = 0.94 ± 0.01, and AU-ROC = 0.93 (0.95 CI [0.91–0.95]) gained more predictive efficiency than other ML-trained algorithms. The factors, including a family history of cancer such as ovary, breast, or colorectal (RI = 0.38), menopausal age (RI = 0.37), history of chest X-ray (RI = 0.35), personal history of breast cancer (RI = 0.35), and postmenopausal hormone therapy (RI = 0.35) were recognized as the influential predictors for OC based XG-Boost. Appraising the current model comprehensiveness through the data cases of two external clinical centers showed that the XG-Boost with AU-ROC = 0.85 (0.95 CI [0.82–0.89]) and AU-ROC = 0.89 (0.95 CI [0.86–0.93]) obtained pleasant interoperability capability in other clinical environments. Although no study has been conducted on leveraging ML for OC based on risk factors, several studies were performed on a similar topic concerning OC. Lu et al. leveraged the ML algorithms to predict the OC using a Chinese dataset, including 49 predictors of demographics, general chemistry, tumor markers, and routine blood tests belonging to malignant and benign OC cases. The 235 and 114 samples were used to train and test the simple decision tree (DT) algorithm. The constructed algorithm was compared to the LR and risk of ovarian malignancy algorithm (ROMA). The results showed that the DT with AU-ROC = 0.888 gained better capability than LR (AU-ROC = 0.877) and ROMA (AU-ROC = 0.814) [34]. The current study used the risk factors to predict OC, contrary to Lu et al.’s study conducted for malignant and benign cases; the current study devised a screening prediction model for stratifying positive and negative cases.

However, leveraging a more vigorous preventive approach based on risk factors, the current study obtained an interoperable XG-Boost model with AU-ROC = 0.93 (0.95 CI [0.91–0.95]). Ahamad et al. utilized an ML approach fed by clinical data from 349 benign and malignant patients to construct a model for detecting OC in the early stages. Based on various scenarios described by features, the gradient boosting machine (GBM) and light GBM with AU-ROC of 0.82 obtained the best performance using the blood test dataset. RF performed best with an AU-ROC of 0.8 for the general chemistry dataset. Also, the RF and XG-boost gained the best performance of prediction capability with an AU-ROC of 0.86 fed by the OC marker dataset [35]. One study by Ziyambe et al. attempted to leverage the DL approach to predict and diagnose OC through histopathological imaging data. To this end, they used the advanced convolutional neural network (CNN) to stratify the malignant cells from healthy ones. Based on the results, the CNN, with an accuracy of 94% (95.12% and 93.02% for classifying cancerous and healthy cells, respectively), gained favorable performance in this respect [40]. Maria et al. constructed ML models to classify OC tumors using a biomarker dataset. Six celebrated algorithms, including linear discriminant analysis (LDA), LR, DT, Naïve Bayes(NB), KNN, and SVM, were leveraged to this aim. All ML algorithms obtained pleasant performance with more than 98% accuracy [41]. Also, in several studies, ML approaches have been leveraged to predict OC survival to give physicians better insight into the situation of OC patients [42, 43]. Our study contribution is introducing preventive solutions through screening the high-risk groups of women concerning OC assisted with ML. Therefore, this strategy is more effective than previous screening methods in earlier stages by stratifying the benign and malignant OC cases. This method significantly impacts preventing OC and its adverse outcomes and death caused by leveraging risk factors.

## Limitations and future implications

This study lacks in some aspects, including using the retrospective approach based on the data of six clinical centers that may affect the predictive capability of the ML algorithms. Some influential determinants concerning OC risk prediction may not be considered, influencing the predictive ability of the models in the current study. Some lost data associated with OC cases were embedded using the imputation method, influencing the generalizability. For future studies, we recommend using more numbers of data for stratification, preferably using the national registry in this respect. Leveraging the mining process in this way has a significant impact on the comprehensiveness of the ML prediction model to stratify OC. However, by leveraging the national registry, the interoperability of the ML model would be increased in the conditions that do not have the registry, using more factors affecting the stratification. We also suggest using actual data instead of the imputation process as much as possible to assure more generalizability of the models. In the current study, we utilized the selected ML algorithms for OC risk stratification. Using various simple and ensemble ML algorithms is also recommended for prediction purposes. Also, we recommend testing the prediction ability of the ML models by the external data belonging to more clinical settings for a better perception of the models' interoperability as possible.

## Conclusion

In the current study, we aimed to construct a novel screening strategy for OC using risk factors and the contribution of ML approaches. We utilized the binary logistic regression as MLR and ML algorithms to select the best predictors affecting OC prediction and develop the prediction model. Based on the results of the current study, the XG-Boost with PPV = 0.94 ± 0.015, NPV = 0.93 ± 0.005, sensitivity = 0.93 ± 0.019, specificity = 0.95 ± 0.002, accuracy = 0.94 ± 0.008, and F-Score = 0.94 ± 0.01, and AU-ROC = 0.93 (0.95 CI [0.91–0.95]) was recognized as the optimal ML algorithm for predicting the OC risk. Based on the current study, the ML approach obtained effective prediction capability for OC. The generalizability testing of our models based on external data cases indicated external AU-ROC of AU-ROC = 0.85 (0.95 CI [0.82–0.89]) and AU-ROC = 0.89 (0.95 CI [0.86–0.93]) for XG-Boost is in two other clinical settings. Other studies focused on screening the malignant and benign types of OC by ML approaches based on clinical data.

Due to the progressive nature of the OC disease, screening suspicious women concerning OC in this way may affect the prognosis of the patients and diminish the efficiency of the various treatment plans. This study introduced a novel screening way for screening OC patients based on risk factors. According to the achievement of this study, the knowledge extracted from the XG-Boost model can be leveraged for developing intelligent systems to screen suspicious women concerning OC based on risk factors. In this way, the high-risk group of women can be identified based on the essential factors influencing the OC. Hence, the efficiency of various preventive strategies for high-risk OC groups would be generated and enhanced. The screening strategy, in this way, can propel the treatment of suspicious people regarding OC to less interventional approaches by identifying the high-risk OC women in a timely manner based on appraising various risk factors. It not only improves the treatment solution for high-risk people and introduces the best treatment and preventive strategy by care providers, but also diminishes the cost of clinical care by introducing more efficient treatment at the community level. Also, identifying the high-risk OC groups at the community level can assist the clinical research on enhancing the solutions for preventing OC.

## Methods

### Study design

This data-driven study, as a retrospective approach, was conducted in five phases. First, after gaining insight into the topic, we determined our study population and attempted to collect appropriate data describing it to achieve our aim. In this respect, we used one integrated electronic database. Second, we prepared our database to advance data quality using various preprocessing methods, such as excluding records or features with missing data more than a specific limit, replacing lost values for records with low-rate missing values, and eliminating the irrelevant features describing samples. In the next phase, we leveraged chosen ML algorithms to build prediction models for OC through data fed. The K-fold cross-validation strategy was used to measure and assess the algorithms’ performance efficiency. This way, through various performance indicators, we obtained the best-performing ML-trained algorithms to achieve the aim of the current study. Finally, we leveraged data cases from external clinical settings to investigate the comprehensiveness of our prediction model for screening OC.

### Study population

In this study, the population was 1516 suspicious OC women referred to six clinical centers in Sari city of Mazandaran Province associated with gynecological cancers to screen themselves from 2015 to 2019. The physician received conclusive positive or negative OC results through various services such as CA-125 blood test, transvaginal ultrasonography, CT-Scan, biopsy, or a mixture. Among 1516 cases, their information was concentrated in one electronic database; 713 and 803 were associated with positive and negative OC cases, respectively.

### Features and outcome variables

The outcome variable was the OC diagnosis, consisting of two positive and negative diagnostic results. There were 26 input features in the database as OC risk predictors, including age, body mass index (BMI), blood group, race, menopausal age, postmenopausal hormone therapy, endometriosis, history of nonpregnancy, family history of ovarian, breast, or colorectal cancer, family cancer syndrome, fertility treatment use, having breast cancer, history of pregnancy and breastfeeding before age 26, history of the ovarian polycystic syndrome (PCOS), history of chest X-ray, smoking, alcohol consumption, particular food consumption, such as fried foods, whole milk, and trans fats, history of exposure to mutagenic or chemical substances, high red meat consumption, vegetable consumption, fruit consumption, high consumption of coffee, aspirin use, history of hysterectomy, and oral contraceptive pill use.

### Preprocessing database

Based on our OC diagnostic dataset, the three-step process was performed in the current study to prepare our database for further analysis. First, we investigated the sample regarding redundancy induced by data integration. In this situation, the redundant cases were excluded from the study. Second, we reviewed the dataset in terms of existing lost data associated with features of samples. We dealt with this situation in two ways: first, samples with more than 5% of missing values were excluded from the study, and second, for the conditions with less than 5%, we used the imputation process through the K-nearest neighborhood (KNN) algorithm with a specific amount of *K*. In this way, we replaced the missing values using the values that existed in most similar cases with *K =* 1, 3, 5, and more. Third, we leveraged the feature selection to obtain the more relevant features for the training process to construct predictive models. Choosing more critical features before the ML process could assist us in putting aside noisy features, decreasing calculation time, promoting learning performance, and facilitating the perception of data and learning models [44, 45]. To get the most important factors associated with OC prediction, we used the multi-variable logistic regression (MLR) and investigated the correlation of predictors in this regard. The *P* < 0.05 was considered a significant statistical level.

### Model development and hyperparameters tuning

After preparing the database, we developed prediction models using ML algorithms. In this respect, the XG-Boost, Random Forest (RF), J-48, support vector machine (SVM), KNN, and artificial neural network (ANN) were leveraged as the most chosen and celebrated algorithms leveraged in previous studies with high-performing in the Weka V 3.9 environment to achieve the prediction aims. We used the best-tuned hyperparameters for each algorithm through the grid search method to get the high-performing ML-trained algorithm. This way, the several hyperparameter combinations are leveraged when reaching the minimum error during the ML process. We used the K (*K =* 10) fold cross-validation technique to gauge and evaluate the algorithms’ performance. In this method, the initial database is split into *K =* 10 folds, in which one section is used to test aims and others for training the algorithms, recurring *K =* 10 epochs. The average error rate of each algorithm in *K =* 10 repetition is considered the algorithm's error rate. Also, to observe the proportion of selected sample numbers having positive and negative diagnosis class labels, we used the stratified type of *K =* tenfold cross-validation to assure more comprehensiveness of ML algorithms’ performance.

### Performance evaluation of selected ML algorithms

We used various performance criteria to achieve the best performance efficiency via measuring, comparing, and assessing the ML-trained algorithms to predict the risk of OC. Hence, we leveraged positive predictive value (PPV), negative predictive value (NPV), sensitivity, specificity, accuracy, and F-Score to measure the performance of ML-trained algorithms as their favorable predictive capability gained in other biomedical research [46–49]. The (true positive) TP and (true negative) TN indicate positive and negative OC diagnoses cases correctly categorized by the models. (False negative) FN and (false positive) FP are equal to these cases incorrectly classified. To assess and contrast the capability of ML algorithms concerning OC prediction effectiveness, we utilized the area under the receiver operator characteristic curve (AU-ROC) of learned algorithms.

$$PPV = \frac{TP}{{TP + FP}},$$

$$NPV = \frac{TN}{{TN + FN}},$$

$$Specificity = \frac{TN}{{TN + FP}},$$

$$Sensitivity = \frac{TP}{{TP + FN}},$$

$$Accuracy = \frac{TP + TN}{{TP + FN + FP + TN}},$$

$$F - Score = \frac{TP}{{TP + \frac{1}{2}(FN + FP)}}.$$

### Evaluating the generalizability nature of the developed prediction model

We used data cases from external clinical settings to assess the interoperability of the current prediction model. In this respect, we used the data from two clinical centers in Tehran City and evaluated our best-performing prediction model’s capability to classify these external data cases. We used 83 and 98 OC cases from these two clinical centers and measured the TP, FP, FN, and TN in this respect. Also, the AU-ROC of the model in two states of internal and external states was utilized. Internal state points to the AU-ROC of the model, which resulted in the current study using six internal clinical settings. On the contrary, the external mode denotes the AU-ROC of our best-performing prediction model when using the data of two external clinical centers. We compared the AU-ROC of our model in these two states to perceive the comprehensiveness and usability of our prediction model for OC in other settings.

## Data Availability

The datasets generated and/or analyzed during the current study are not publicly available due to the privacy concerns of research committee but are available from the corresponding author on reasonable request.

## References

[1] Momenimovahed Z, Tiznobaik A, Taheri S, Salehiniya H (2019). Ovarian cancer in the world: epidemiology and risk factors. Int J Womens Health.

[2] Gaona-Luviano P, Medina-Gaona LA, Magaña-Pérez K (2020). Epidemiology of ovarian cancer. Chin Clin Oncol.

[3] La Vecchia C (2017). Ovarian cancer epidemiology and risk factors. Eur J Cancer Prev.

[4] Orr B, Edwards RP (2018). Diagnosis and treatment of ovarian cancer. Hematol Oncol Clin North Am.

[5] Lisio M-A, Fu L, Goyeneche A, Gao Z-h, Telleria C (2019). High-grade serous ovarian cancer: basic sciences, clinical and therapeutic standpoints. Int J Mol Sci.

[6] Sun S-n, Hu S, Shang Y-p, Li L-y, Zhou H, Chen J-s (2019). Relevance function of microRNA-708 in the pathogenesis of cancer. Cell Signal.

[7] Stewart C, Ralyea C, Lockwood S (2019). Ovarian cancer: an integrated review. Semin Oncol Nurs.

[8] Torre LA, Trabert B, DeSantis CE, Miller KD, Samimi G, Runowicz CD (2018). Ovarian cancer statistics, 2018. CA Cancer J Clin.

[9] Huang J, Chan WC, Ngai CH, Lok V, Zhang L, Lucero-Prisno DE (2022). Worldwide burden, risk factors, and temporal trends of ovarian cancer: a global study. Cancers.

[10] Permuth-Wey J, Sellers TA, Verma M (2009). Epidemiology of ovarian cancer. Cancer epidemiology: modifiable factors.

[11] Malvezzi M, Carioli G, Rodriguez T, Negri E, La Vecchia C (2016). Global trends and predictions in ovarian cancer mortality. Ann Oncol.

[12] Webb PM, Jordan SJ (2017). Epidemiology of epithelial ovarian cancer. Best Pract Res Clin Obstet Gynaecol.

[13] Zhang Y, Luo G, Li M, Guo P, Xiao Y, Ji H (2019). Global patterns and trends in ovarian cancer incidence: age, period and birth cohort analysis. BMC Cancer.

[14] Mohammadian M, Ghafari M, Khosravi B, Salehiniya H, Aryaie M, Bakeshei FA (2017). Variations in the incidence and mortality of ovarian cancer and their relationship with the human development index in European Countries in 2012. Biomed Res Ther.

[15] Maryam B, Fatemeh S, Nourossadat K, Saeideh N, Giti O (2022). Women's awareness of ovarian cancer risk factors and symptoms in Western Iran in 2020–2021. BMC Womens Health.

[16] Sharifian A, Pourhoseingholi MA, Norouzinia M, Vahedi M (2014). Ovarian cancer in Iranian women, a trend analysis of mortality and incidence. Asian Pac J Cancer Prev.

[17] Akbari A, Looha MA, Moradi A, Akbari ME (2023). Ovarian cancer in Iran: national based study. Iran J Public Health.

[18] Šekerija M, Čukelj P (2015). Epidemiology of ovarian cancer in Croatia. Libri Oncol.

[19] US Preventive Services Task Force (2018). Screening for ovarian cancer: us preventive services task force recommendation statement. JAMA.

[20] Ongsulee P, Chotchaung V, Bamrungsi E, Rodcheewit T, Ongsulee P, Chotchaung V, Bamrungsi E, Rodcheewit T (2018). Big data, predictive analytics and machine learning. 2018 16th international conference on ICT and knowledge engineering (ICT&KE); 2018 21–23 Nov.

[21] Lalmuanawma S, Hussain J, Chhakchhuak L (2020). Applications of machine learning and artificial intelligence for Covid-19 (SARS-CoV-2) pandemic: a review. Chaos Solitons Fractals.

[22] Bertsimas D, Wiberg H (2020). Machine learning in oncology: methods, applications, and challenges. JCO Clin Cancer Inform.

[23] Stark GF, Hart GR, Nartowt BJ, Deng J (2019). Predicting breast cancer risk using personal health data and machine learning models. PLoS ONE.

[24] Ming C, Viassolo V, Probst-Hensch N, Chappuis PO, Dinov ID, Katapodi MC (2019). Machine learning techniques for personalized breast cancer risk prediction: comparison with the BCRAT and BOADICEA models. Breast Cancer Res.

[25] Akbar S, Hayat M (2018). iMethyl-STTNC: identification of N6-methyladenosine sites by extending the idea of SAAC into Chou's PseAAC to formulate RNA sequences. J Theor Biol.

[26] Akbar S, Hayat M, Iqbal M, Jan MA (2017). iACP-GAEnsC: Evolutionary genetic algorithm based ensemble classification of anticancer peptides by utilizing hybrid feature space. Artif Intell Med.

[27] Ali F, Ahmed S, Swati ZNK, Akbar S (2019). DP-BINDER: machine learning model for prediction of DNA-binding proteins by fusing evolutionary and physicochemical information. J Comput Aided Mol Des.

[28] Akbar S, Khan S, Ali F, Hayat M, Qasim M, Gul S (2020). iHBP-DeepPSSM: Identifying hormone binding proteins using PsePSSM based evolutionary features and deep learning approach. Chemom Intell Lab Syst.

[29] Akbar S, Ahmad A, Hayat M, Rehman AU, Khan S, Ali F (2021). iAtbP-Hyb-EnC: prediction of antitubercular peptides via heterogeneous feature representation and genetic algorithm based ensemble learning model. Comput Biol Med.

[30] Akbar S, Hayat M, Tahir M, Khan S, Alarfaj FK (2022). cACP-DeepGram: Classification of anticancer peptides via deep neural network and skip-gram-based word embedding model. Artif Intell Med.

[31] Shinde PP, Shah S, Shinde PP, Shah S (2018). A review of machine learning and deep learning applications. 2018 fourth international conference on computing communication control and automation (ICCUBEA); 2018 16–18 Aug.

[32] Atitallah SB, Driss M, Boulila W, Ghézala HB (2020). Leveraging deep learning and IoT big data analytics to support the smart cities development: review and future directions. Comput Sci Rev.

[33] Gong X, Zheng B, Xu G, Chen H, Chen C (2021). Application of machine learning approaches to predict the 5-year survival status of patients with esophageal cancer. J Thorac Dis.

[34] Lu M, Fan Z, Xu B, Chen L, Zheng X, Li J (2020). Using machine learning to predict ovarian cancer. Int J Med Inform.

[35] Ahamad MM, Aktar S, Uddin MJ, Rahman T, Alyami SA, Al-Ashhab S (2022). Early-stage detection of ovarian cancer based on clinical data using machine learning approaches. J Pers Med.

[36] Mohammad Reza A, Leila E, Morteza A, Nahid M, Saeed J, Raoof N (2022). Machine Learning-Based Clinical Decision Support System for automatic diagnosis of COVID-19 based on the routine blood test. J Biostat Epidemiol.

[37] Shanbehzadeh M, Nopour R, Erfannia L, Amraei M, Mehrabi N, Mashoufi M (2022). Comparing data mining algorithms for breast cancer diagnosis. Shiraz E Med J.

[38] Nopour R, Erfannia L, Mehrabi N, Mashoufi M, Mahdavi A, Shanbehzadeh M (2022). Comparison of two statistical models for predicting mortality in COVID-19 patients in Iran. Shiraz E Med J.

[39] Raoof N, Mostafa S, Nahid M (2022). Developing an intelligent tool for breast cancer prognosis using artificial neural network. Acta Med Iran.

[40] Ziyambe B, Yahya A, Mushiri T, Tariq MU, Abbas Q, Babar M (2023). A deep learning framework for the prediction and diagnosis of ovarian cancer in pre-and post-menopausal women. Diagnostics.

[41] Maria HH, Jossy AM, Malarvizhi S (2022). A machine learning approach for classification of ovarian tumours.

[42] Paik ES, Lee J-W, Park J-Y, Kim J-H, Kim M, Kim T-J (2019). Prediction of survival outcomes in patients with epithelial ovarian cancer using machine learning methods. J Gynecol Oncol..

[43] Sorayaie Azar A, Babaei Rikan S, Naemi A, Bagherzadeh Mohasefi J, Pirnejad H, Bagherzadeh Mohasefi M (2022). Application of machine learning techniques for predicting survival in ovarian cancer. BMC Med Inform Decis Mak.

[44] Cai J, Luo J, Wang S, Yang S (2018). Feature selection in machine learning: a new perspective. Neurocomputing.

[45] Ang JC, Mirzal A, Haron H, Hamed HNA (2015). Supervised, unsupervised, and semi-supervised feature selection: a review on gene selection. IEEE ACM Trans Comput Biol Bioinf.

[46] Kha QH, Ho QT, Le NQK (2022). Identifying SNARE proteins using an alignment-free method based on multiscan convolutional neural network and PSSM profiles. J Chem Inf Model.

[47] Le NQK, Ho QT, Nguyen VN, Chang JS (2022). BERT-promoter: an improved sequence-based predictor of DNA promoter using BERT pre-trained model and SHAP feature selection. Comput Biol Chem.

[48] Nopour R, Mashoufi M, Amraei M, Mehrabi N, Mohammadnia A, Mahdavi A (2022). Performance analysis of selected decision tree algorithms for predicting drug adverse reaction among COVID-19 hospitalized patients. J Med Chem Sci.

[49] Nopour R, Shanbehzadeh M, Kazemi-Arpanahi H (2021). Developing a clinical decision support system based on the fuzzy logic and decision tree to predict colorectal cancer. Med J Islam Repub Iran.

